# Resampling Point Clouds Using Series of Local Triangulations

**DOI:** 10.3390/jimaging11020049

**Published:** 2025-02-08

**Authors:** Vijai Kumar Suriyababu, Cornelis Vuik, Matthias Möller

**Affiliations:** Delft Institute of Applied Mathematics, Delft University of Technology, Mekelweg 4, 2628 CD Delft, The Netherlands; c.vuik@tudelft.nl (C.V.); m.moller@tudelft.nl (M.M.)

**Keywords:** point-cloud resampling, surface reconstruction, feature preservation

## Abstract

The increasing reliance on 3D scanning and meshless methods highlights the need for algorithms optimized for point-cloud geometry representations in CAE simulations. While voxel-based binning methods are simple, they often compromise geometry and topology, particularly with coarse voxelizations. We propose an algorithm based on a Series of Local Triangulations (SOLT) as an intermediate representation for point clouds, enabling efficient upsampling and downsampling. This robust and straightforward approach preserves the integrity of point clouds, ensuring resampling without feature loss or topological distortions. The proposed techniques integrate seamlessly into existing engineering workflows, avoiding complex optimization or machine learning methods while delivering reliable, high-quality results for a large number of examples. Resampled point clouds produced by our method can be directly used for solving PDEs or as input for surface reconstruction algorithms. We demonstrate the effectiveness of this approach with examples from mechanically sampled point clouds and real-world 3D scans.

## 1. Introduction

Triangular meshes have long been the standard representation for discrete surfaces in computational geometry. However, point clouds have emerged as a viable alternative, particularly in scenarios where generating a surface mesh is challenging [1]. Techniques such as smoothed-particle hydrodynamics (SPH) and radial basis function-finite differences (RBF-FD) [2] use tree-based representations [3] to establish connectivity in point clouds, offering advantages over traditional mesh-based methods for specific applications. Furthermore, advances in point-based rendering, driven by neural networks [4], highlight the growing importance of point clouds. Innovations in 3D scanning and LiDAR have cemented their role as a reliable representation of complex 3D geometries.

### 1.1. Related Work (Algorithmic Approaches)

Point clouds can be oriented or unoriented depending on their source. Establishing consistent orientations in unoriented point clouds often requires specialized algorithms [5]. Additionally, point clouds must adhere to specific distribution patterns or maintain a desired resolution to ensure their effective use. While various methods have been developed for resampling point clouds [6,7,8,9,10], most approaches focus exclusively on either upsampling or downsampling. We make a comparison against some of these works in our Numerical Experiments section.

Voxelization is a common intermediate representation used in resampling workflows. For instance, Chenlei et al. [8] constructed occupancy grids for point clouds and resampled them by averaging local voxel neighborhoods. However, voxelization often introduces artifacts that necessitate additional projection or optimization steps. While such artifacts may be tolerable for certain applications, CAE workflows typically demand highly uniform point-cloud distributions [3].

### 1.2. Related Work (Learning-Based Approaches)

Learning-based methods have emerged as powerful tools for point-cloud processing, leveraging deep learning architectures to tackle tasks such as denoising, resampling, and reconstruction [11]. For instance, Zhao et al. [12] proposed a multi-task learning network for LiDAR point-cloud preprocessing, incorporating denoising, segmentation, and completion branches. Similarly, Zhao et al. [13] introduced the ICDDPM model for image-conditioned single-view reconstruction, achieving state-of-the-art results on datasets like ShapeNet and PASCAL3D+. Other works, such as those by Wu et al. [14], Chen et al. [15], and Rong et al. [16], focus on advanced resampling and upsampling techniques, demonstrating impressive performance across diverse non-CAE datasets.

Despite their effectiveness, these learning-based methods are not explicitly tailored for computer-aided engineering (CAE) applications. For completeness, we still include comparisons with select learning-based methods in our Numerical Experiments section, showcasing the strengths of our approach.

### 1.3. Our Approach

In contrast to the aforementioned methods, we propose a novel algorithm leveraging an intermediate representation termed the Series of Local Triangulations (SOLT). By constructing and refining local Delaunay triangulations, SOLT avoids the artifacts associated with voxelization while ensuring uniformity and topology preservation. Unlike deep learning approaches, SOLT does not rely on pre-trained models, making it lightweight and suitable for seamless integration into CAE workflows.

### 1.4. Contributions

The key contributions of this work are as follows:

1. A novel algorithm, SOLT, enabling efficient point-cloud upsampling and downsampling without artifacts or topology loss.
2. A demonstration of SOLT’s robustness across a variety of inputs, including mechanically sampled point clouds and real-world 3D scans.
3. A comprehensive evaluation of SOLT against state-of-the-art methods, highlighting its accuracy, computational efficiency, and fidelity.
4. The quantitative and qualitative results, illustrating SOLT’s versatility in CAE applications.

## 2. Methodology

**Figure 1:**
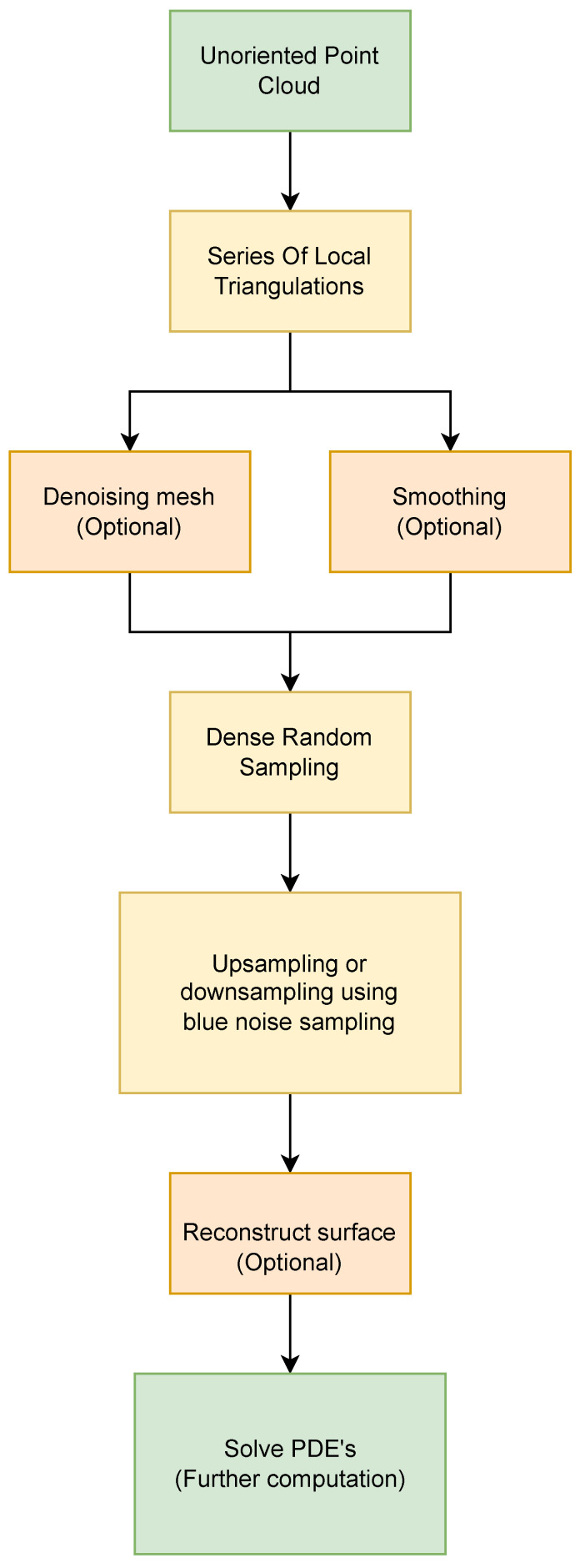
Workflow of the overall methodology. Optional modules are highlighted in light orange.

Figure 1 illustrates the point-resampling workflow. Steps such as denoising and smoothing are optional and depend on the characteristics of the input point cloud. The methodologies for these steps are detailed in [17]. These filters are handy for handling noise or perturbations introduced by data-collection sources, such as LiDAR or 3D scanners. Notably, our approach does not modify the point cloud directly; adjustments are applied to the intermediate mesh representation described in the following sections.

### 2.1. Series of Local Triangulations (SOLT)

The Series of Local Triangulations (SOLT) technique represents point-cloud data by constructing localized triangulations around individual points. It uses distance and geometric parameters (tangent coordinates) to identify nearby points and computes a characteristic length scale for each local neighbourhood, ensuring adaptability to varying point densities. Heuristics are applied to handle problematic configurations, maintaining stability and preserving geometry. Points are sorted to enable consistent local Delaunay triangulations, followed by iterative refinements. The resulting local triangulations are merged into a global triangulation by de-duplicating indices.

#### 2.1.1. Nearest Point Selection for Local Triangulation

A key parameter in the SOLT algorithm is the search radius *r*, which defines the neighbourhood of each point. The neighborhood S(p) for each point *p* is mathematically defined as:

S(p)={q∈PointCloud|∥q−p∥≤r,and∥T(q)−T(p)∥≤ϵ},

where:

- *r*: Search radius (default: average point-to-point distance),
- T(p) and T(q): Tangent coordinate vectors at *p* and *q*,
- ϵ: Threshold for tangent coordinate similarity.

Alternatively, a fixed number of nearest points (*k*) can be used instead of a radius. The radius *r* is computed as:

$$r=\frac{1}{n}\sum_{i=1}^{n}∥p_{i}-p_{j}∥,\;\forall p_{j}\in \mathrm{NearestNeighbors}\left(p_{i}\right),$$

where *n* is the total number of points in the point cloud.

#### 2.1.2. Mesh Quality and Robustness

The SOLT mesh is an intermediate representation. Issues such as self-intersections or non-manifold edges do not affect the final resampling results. The SOLT representation supports the following operations:

- Random sampling,
- Blue noise sampling (a refinement of random sampling),
- Feature distance field computations.

#### 2.1.3. Circumcircle Criterion for Delaunay Triangulation

Delaunay triangulation ensures that no point lies inside the circumcircle of any triangle. For a triangle $▵(p_{1},p_{2},p_{3})$, the circumcircle condition is:

$$\mathrm{CircumcircleCondition}:\;∥q-c∥>r_{c},\;\forall q\notin \left\{p_{1},p_{2},p_{3}\right\},$$

where:

- *c*: Circumcenter of $▵(p_{1},p_{2},p_{3})$,
- $r_{c}$: Circumradius of $▵(p_{1},p_{2},p_{3})$,
- *q*: Any point outside the triangle.

Edges violating this condition are flipped to restore the Delaunay property.

### 2.2. Examples and Comparisons

**Figure 2:**
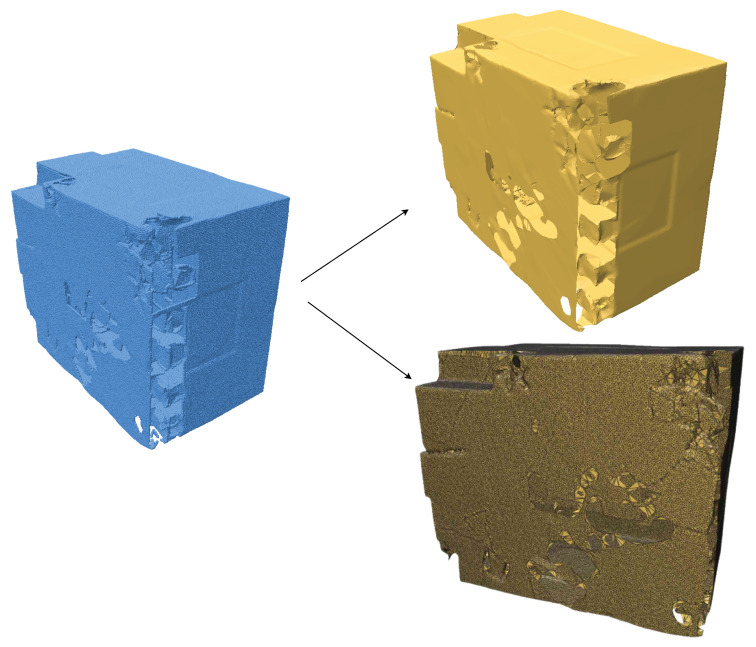
Point cloud (blue) converted to a Series of Local Triangulations (SOLT) representation. The SOLT is shown in yellow, both with and without edges.

A sample of a building point cloud using the SOLT representation is shown in Figure 2. This representation highlights the effectiveness of SOLT in capturing local geometric details and preserving key structural features. To further demonstrate the robustness of the SOLT method, we present additional examples, including comparisons with other popular surface reconstruction techniques, such as the Ball-Pivoting Algorithm (BPA) and Poisson reconstruction.

**Figure 3:**
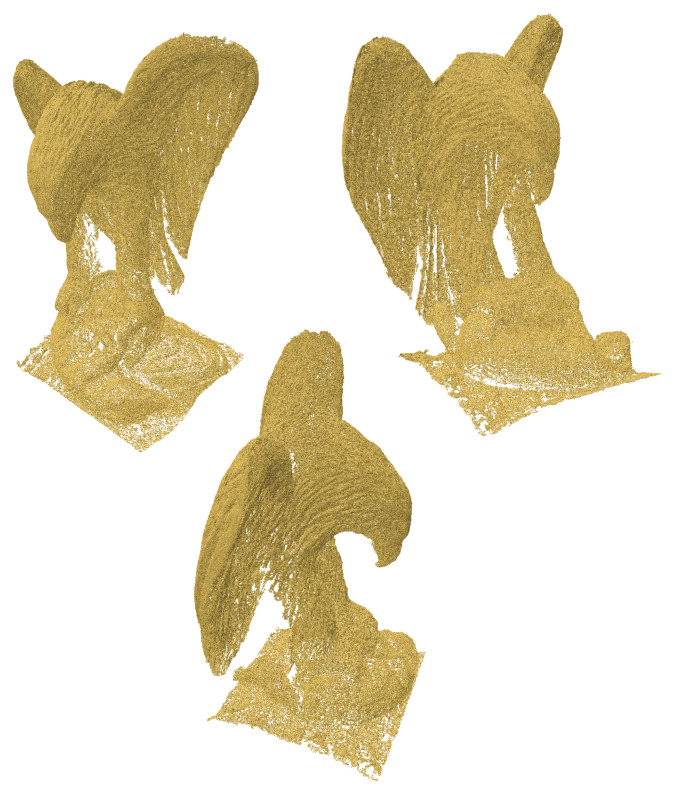
Different views of an eagle point cloud (from Open3D’s datasets [18]). The point cloud (796,825 points) contains intricate features, making it an excellent candidate for evaluating reconstruction algorithms.

**Figure 4:**
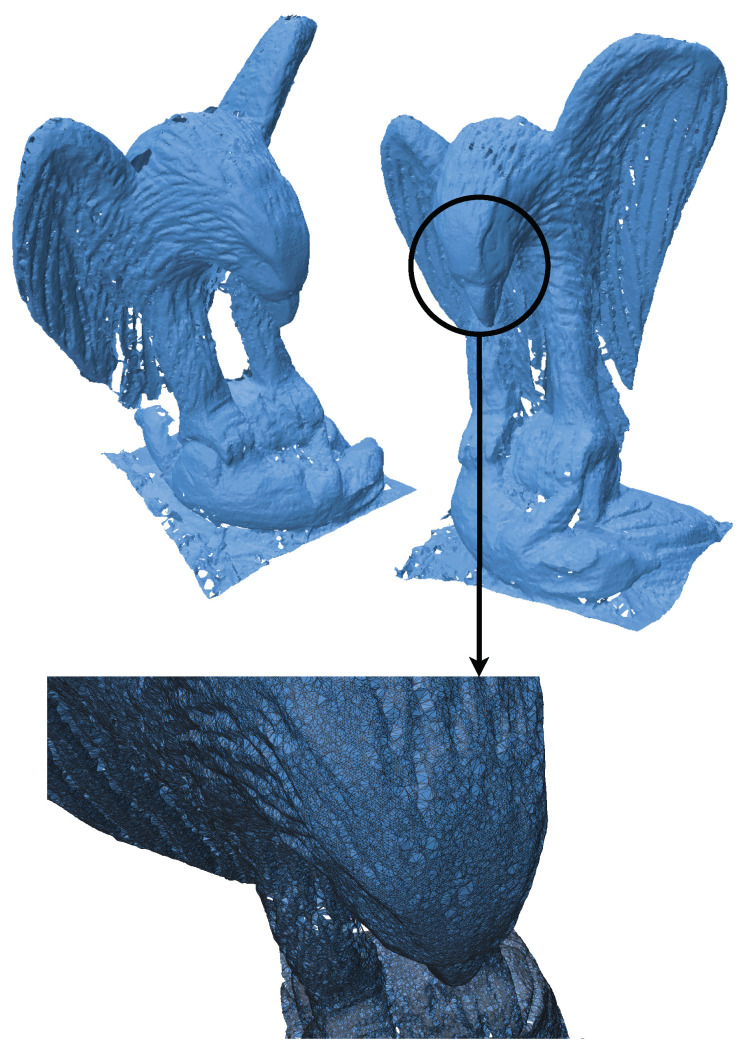
SOLT reconstruction of the eagle point cloud (Time taken: 35.8 s). The SOLT algorithm effectively captures the intricate features of the point cloud while being computationally efficient.

**Figure 5:**
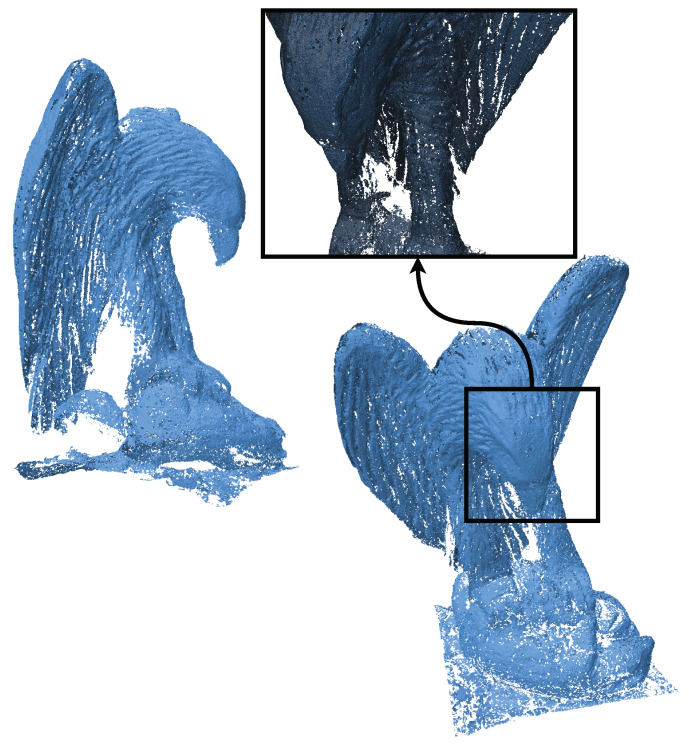
BPA reconstruction of the eagle point cloud (Time taken: 26.91 min). This method is 62 times slower than the SOLT algorithm, achieving a similar reconstruction quality.

**Figure 6:**
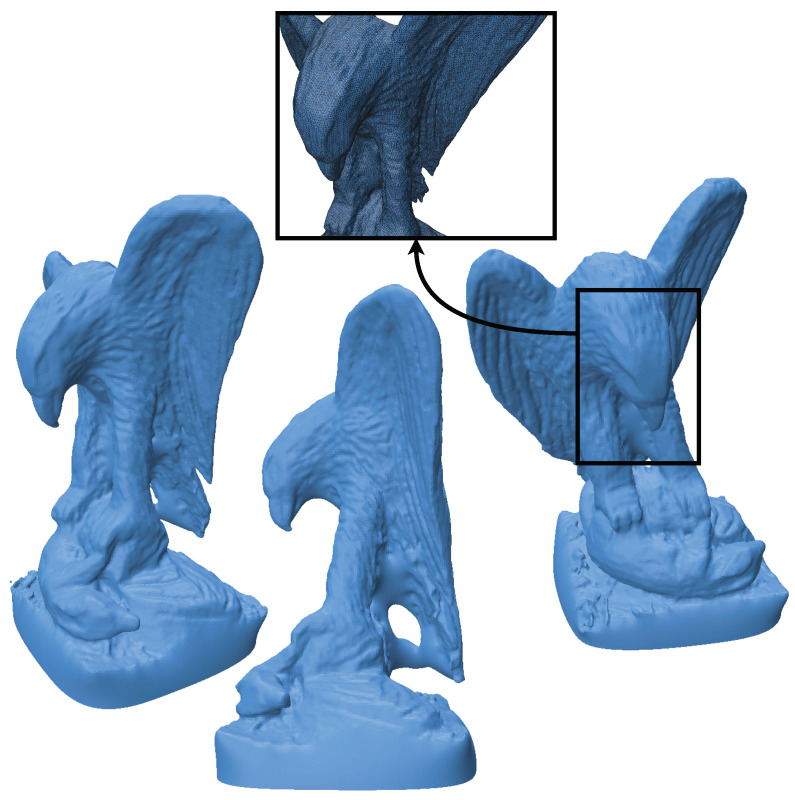
Poisson reconstruction of the eagle point cloud (Time taken: 87.9 s). This method is 2.46 times slower than the SOLT algorithm, achieving comparable quality.

The eagle point cloud, sourced from Open3D’s datasets [18], is used to evaluate the performance and efficiency of our algorithm. Figure 3, Figure 4, Figure 5 and Figure 6 illustrate the input point cloud and the reconstructions generated by SOLT, BPA, and Poisson reconstruction approaches.

#### 2.2.1. SOLT Reconstruction

Figure 4 shows the SOLT reconstruction of the eagle point cloud, which took 35.8 s to complete. The SOLT algorithm effectively retains the eagle’s intricate details and overall geometry while maintaining computational efficiency. It can be clearly seen that the topology of the mesh remains intact. This performance demonstrates the algorithm’s suitability for real-time applications or scenarios where computational resources are limited.

#### 2.2.2. BPA Reconstruction

The Ball-Pivoting Algorithm (BPA) reconstruction of the eagle point cloud (Figure 5) took significantly longer, completing in 26.91 min. While BPA successfully reconstructs the geometry, it is approximately 62 times slower than SOLT, highlighting its inefficiency for large or complex point clouds. This comparison underscores the advantage of SOLT in handling high-resolution datasets efficiently.

#### 2.2.3. Poisson Reconstruction

The Poisson reconstruction approach (Figure 6) was completed in 87.9 s for a comparable level of detail. While it is approximately 2.46 times slower than SOLT, it is significantly faster than BPA. Poisson reconstruction balances computational speed and detail preservation but has notable limitations.

One key drawback of Poisson reconstruction is its tendency to over-smooth fine details, which can result in the loss of sharp features. Additionally, the method operates as a global solver, offering limited control over local geometric properties. While this global approach ensures consistency, it may inadvertently alter the genus or modify essential features of the input geometry. These characteristics make Poisson reconstruction less suitable for tasks like mesh resampling, where preserving topology and fine details is critical. Despite its strengths as a reconstruction algorithm, these drawbacks limit its attractiveness for applications requiring high fidelity to the original input.

### 2.3. Discussion

The comparative analysis of these reconstruction techniques highlights the SOLT algorithm’s distinct advantages. It not only outperforms BPA by a substantial margin in terms of computational efficiency but also achieves comparable reconstruction quality to both BPA and Poisson methods.

- Efficiency: SOLT’s 35.8-second runtime demonstrates its computational advantage, making it suitable for time-sensitive applications.
- Quality: The SOLT representation accurately captures intricate features such as the eagle’s wings and body structure, maintaining high fidelity to the input point cloud.
- Versatility: While Poisson reconstruction is faster than BPA, SOLT achieves similar quality with significantly lower computational cost, cementing its robustness across diverse use cases.

A more detailed comparison is presented in Table 1, highlighting the SOLT algorithm’s efficiency and robustness for point-cloud resampling and other applications.

#### Summary

The pseudocode in Algorithm 1 is simple and can be easily integrated into geometry processing pipelines. Nicholas Sharp et al. utilized a similar approach to address degenerate meshes in solving PDEs [19]. In prior work, this representation effectively transformed unoriented point clouds for consistent and reliable winding number computations [17]. The SOLT approach delivers results comparable to more complex methods involving PDEs [5,20].
**Algorithm** **1** Series of Local Triangulations (SOLT)1:**function** seriesOfLocalTriangulations2:      **Input:** Unoriented point cloud3:      **Output:** Series of Local Triangulations4:      Initialize the point cloud5:      **for** each point *p* in the point cloud **do**6:            Identify a local neighborhood S(p) of *p* using a distance threshold or tangent space coordinates7:            Determine the characteristic length scale of S(p) as the distance to its farthest neighbor8:            **if** points in S(p) are excessively close or nearly coincident **then**9:                  Adjust their positions to prevent degeneracies10:          **end if**11:          Arrange points in S(p) in a counter-clockwise order for consistency12:          Compute the local Delaunay triangulation of S(p)13:          Refine the triangulation to ensure it adheres to the Delaunay criterion14:    **end for**15:    Merge the local triangulations into a global mesh by eliminating duplicate vertices16:    Optionally, apply smoothing or denoising as described in [17]17:**end function**

### 2.4. Point Resampling

After calculating the intermediate representation described in Section 2.1, the next step involves resampling the points to meet specific application requirements. Point resampling can be tailored to generate a new point cloud with desired characteristics, such as uniform distribution, adherence to distance constraints, or alignment with specific features like sharp edges or curves. Depending on the requirements, different resampling techniques can be employed.

If random sampling is sufficient, the resampling process can use methods that rely on random numbers and triangle areas to generate a new point cloud as outlined in Algorithm 2. However, random sampling may not always be ideal for applications requiring precision or uniformity, as it can lead to uneven distributions or clustering artifacts. For more structured applications, such as upsampling or downsampling, employing more sophisticated techniques like blue noise sampling is preferable.
**Algorithm** **2** Random sampling on SOLT (area-weighted sampling)1:**function** randomSamplingOnSOLT(SOLT, numPoints)2:      **Input:** SOLT (Series of Local Triangulations), numPoints (desired number of points)3:      **Output:** Randomly sampled point cloud4:      Compute the area of each triangle *T* in SOLT5:      Normalize triangle areas to form a cumulative distribution function (CDF)6:      Initialize an empty set *S*7:      **for** each *i* from 1 to numPoints **do**8:            Select a triangle *T* randomly, weighted by its area, using the CDF9:            Generate random barycentric coordinates (u,v,w), where u+v+w=110:          Compute the sampled point *p* as $p=uv_{1}+vv_{2}+wv_{3}$, where $v_{1},v_{2},v_{3}$ are the vertices of *T*11:          Add *p* to *S*12:    **end for**13:    **return** *S*14:**end function**

Blue noise sampling is one of the most effective methods for high-quality resampling, which produces a well-spaced and uniform distribution of points while satisfying user-defined distance criteria. Blue noise patterns minimize visual artifacts and clustering, making them suitable for computational tasks requiring consistent point densities. The work of Robert Bridson [21] describes an efficient and widely adopted approach for generating blue noise distributions.

Point resampling typically begins with a dense random sampling phase based on the SOLT representation. This dense point set is refined into a blue noise-sampled point cloud that preserves desired spacing and uniformity as outlined in Algorithm 3. By leveraging triangle areas and random barycentric sampling, these methods ensure that resampled point clouds respect the application’s geometry and density requirements.
**Algorithm** **3** Blue noise sampling on SOLT (area-weighted sampling with distance constraint)1:**function** blueNoiseSamplingOnSOLT(SOLT, minDistance)2:      **Input:** SOLT (Series of Local Triangulations), minDistance (minimum spacing between points)3:      **Output:** Blue noise-sampled point cloud4:      Compute the area of each triangle *T* in SOLT5:      Normalize triangle areas to form a cumulative distribution function (CDF)6:      Initialize an empty set *S* and a candidate queue *Q*7:      Randomly select an initial triangle $T_{0}$ from the CDF8:      Generate a random point $p_{0}$ within $T_{0}$ using barycentric coordinates and add $p_{0}$ to *S* and *Q*9:      **while** *Q* is not empty **do**10:          Remove a point *p* from *Q*11:          **for** each candidate point *c* generated around *p* **do**12:                Select a triangle $T_{c}$ containing *c*, weighted by area, using the CDF13:                **if** *c* lies within $T_{c}$ and satisfies the minDistance criterion from all points in *S* **then**14:                      Add *c* to *S*15:                      Add *c* to *Q*16:                **end if**17:          **end for**18:     **end while**19:     **return** *S*20:**end function**

When resampling point clouds, additional considerations may include incorporating feature constraints. For example, if the input point cloud includes feature curves or sharp edges, the sampling process can be constrained to ensure that these features are adequately captured in the output. This is particularly relevant in applications with critical geometric fidelity, such as surface reconstruction or finite element analysis.

Furthermore, blue noise sampling can be adapted to handle non-uniform distributions, such as areas requiring higher point density due to localized curvature or features of interest. The algorithm can produce point clouds tailored to specific geometric and application needs by introducing variable density criteria or weight-based sampling.

The application’s requirements should guide the choice of resampling technique, whether it demands random sampling, high-quality blue noise sampling, or feature-aware constraints.

### 2.5. Feature Distance Field and Feature-Preserving Resampling

Preserving features in point-cloud resampling is crucial for applications requiring geometric fidelity. In the existing literature, feature preservation is often overlooked or handled using heuristic-based techniques. To address this, we estimate a distance field from feature points onto the mesh and use it as a constraint during resampling. The generalized signed distance field is computed using the method described in [22], which provides robust estimates even for extremely poor quality triangulations (often the case with SOLT).

This feature distance field D(x) is used to augment the resampling process, ensuring that newly sampled points are sensitive to features. Instead of directly incorporating feature points, we define a distance-based constraint on the sampling process using the field D(x). The distance field captures the proximity of any point in the mesh to the nearest feature, providing a smooth, spatially aware constraint as outlined in Algorithm 4.

During blue noise sampling, the original point cloud $P=\{p_{1},p_{2},\ldots ,p_{n}\}$ is resampled using the feature distance field. A minimum distance *d* is enforced between any two sampled points, while the distance field D(x) is used to modify the sampling distribution. Regions’ near features are sampled more densely based on the field values. Points are sampled only if they satisfy the minimum distance criterion and the constraints imposed by D(x).
**Algorithm** **4** Feature-preserving resampling using feature distance field1:**function** featurePreservingResampling2:      **Input:** Original point cloud *P*, feature distance field D(x)3:      **Output:** Resampled point cloud $P^{\prime }$4:      Estimate seriesOfLocalTriangulations for *P* as per Algorithm 15:      Compute feature distance field D(x) using [22]6:      Initialize $P^{\prime }=\emptyset$7:      Set minimum distance *d*8:      **while** not converged or maximum iterations not reached **do**9:            Generate candidate point $p^{\prime }$ using blue noise sampling10:           **if** $D(p^{\prime })<\mathrm{threshold}$ and $p^{\prime }$ satisfies distance criterion with all existing points in $P^{\prime }$ **then**11:                Add $p^{\prime }$ to $P^{\prime }$12:           **end if**13:     **end while**14:     **return** $P^{\prime }$15:**end function**

**Figure 7:**
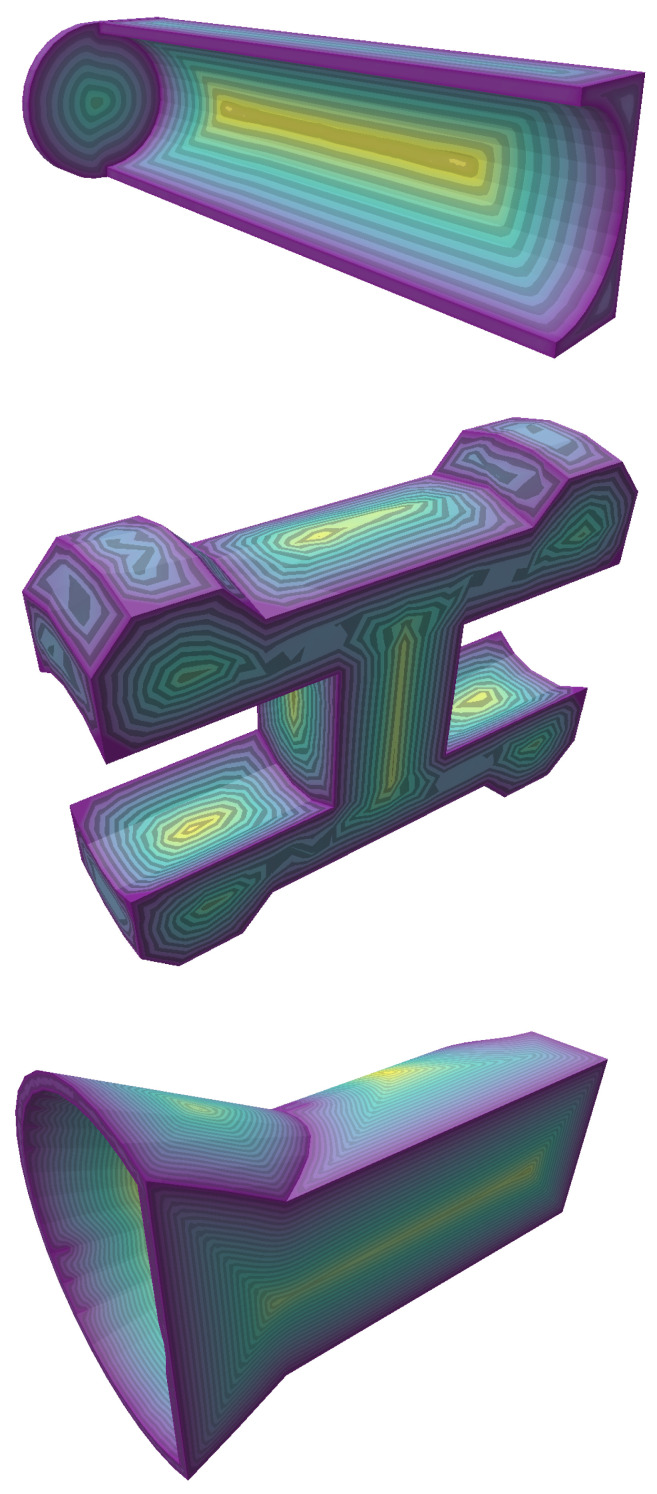
Feature distance fields for selected geometries (purple indicates a distance field value of zero).

Figure 7 illustrates examples of feature distance fields for selected geometries, showcasing how distances from feature curves to the surrounding mesh are computed. These fields guide the resampling process, ensuring that critical features such as sharp edges or curves are preserved in the final point cloud.

By leveraging the feature distance field, our method ensures that resampled point clouds are sensitive to features without directly relying on feature points. This approach maintains geometric fidelity while enabling robust resampling for applications in surface reconstruction, finite element analysis, and other geometry-sensitive domains.

## 3. Numerical Experiments

The algorithm was implemented in C++ and evaluated using datasets including the Waterloo Point Cloud Database [23,24], Thingi10k [25], and SimJEB [26]. The tests were conducted on an Intel i5-8350U laptop with 8 threads and an integrated GPU. These datasets feature diverse geometries such as everyday objects, mechanical components, and intricate high-genus structures, providing a comprehensive evaluation of the algorithm’s performance in feature preservation, noise handling, and topological fidelity.

### 3.1. Quantitative Metrics

To evaluate the robustness of our algorithm, the following quantitative metrics were considered:

1. Chamfer Distance Loss (%): Calculates the average bidirectional distance between two point clouds as a percentage. It is defined as:
  $$\mathrm{Chamfer}\;\mathrm{Loss}\;(\%)=\left(\frac{1}{\left|P\right|}\sum_{p\in P}\min_{q\in Q}{∥p-q∥}^{2}+\frac{1}{\left|Q\right|}\sum_{q\in Q}\min_{p\in P}{∥p-q∥}^{2}\right)\times 100$$
  where *P* and *Q* represent the original and resampled point clouds, respectively.
2. Hausdorff Distance Loss (%): Captures the maximum distance between the closest points of two point clouds, expressed as a percentage. It is defined as:
  $$\mathrm{Hausdorff}\;\mathrm{Loss}\;(\%)=\left(\max\left\{\underset{p\in P}{\sup}\underset{q\in Q}{\inf}∥p-q∥,\underset{q\in Q}{\sup}\underset{p\in P}{\inf}∥p-q∥\right\}\right)\times 100$$
3. Uniformity Index (%): Evaluates the evenness of point distribution across the resampled point cloud:
  $$\mathrm{Uniformity}\;\mathrm{Index}\;(\%)=\left(1-\frac{\sigma_{\mathrm{nn}}}{\mu_{\mathrm{nn}}}\right)\times 100$$
  where $\mu_{\mathrm{nn}}$ and $\sigma_{\mathrm{nn}}$ are the mean and standard deviation of nearest-neighbor distances. Higher percentages indicate more consistent point spacing.
4. Volume Preservation Error (%): Quantifies the percentage difference in volume between the original and resampled meshes:
  $$\mathrm{Volume}\;\mathrm{Preservation}\;\mathrm{Error}\;(\%)=\frac{|V_{\mathrm{original}}-V_{\mathrm{resampled}}|}{V_{\mathrm{original}}}\times 100$$
  where $V_{\mathrm{original}}$ and $V_{\mathrm{resampled}}$ are the original and resampled volumes.
5. Computational Time (s): Measures the time taken by the algorithm to process and resample a point cloud:
  $$T_{\mathrm{compute}}=t_{\mathrm{end}}-t_{\mathrm{start}}$$
6. Compression Ratio: Assesses the reduction in data size during resampling:
  $$\mathrm{Compression}\;\mathrm{Ratio}=\frac{{\mathrm{Size}}_{\mathrm{original}}}{{\mathrm{Size}}_{\mathrm{resampled}}}$$

### 3.2. Smooth Geometries

**Figure 8:**
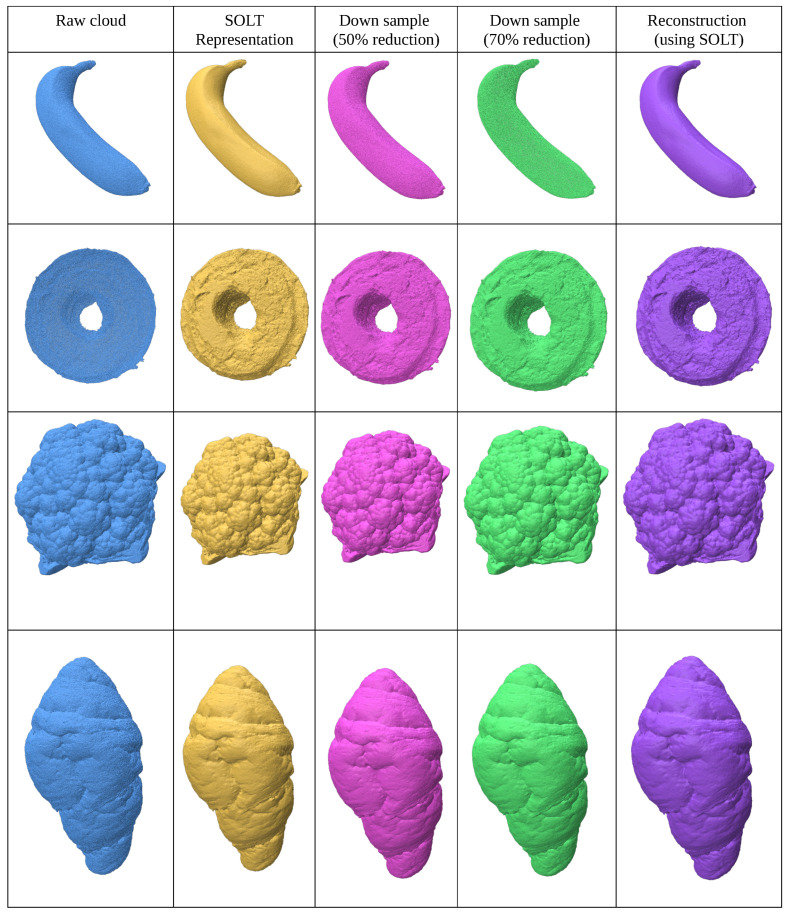
Point cloud (blue) meshed using SOLT (yellow), downsampled in two stages (pink and green), and reconstructed using the SOLT representation (purple).

This experiment focuses on resampling point clouds from smooth geometries with low genus, using the Waterloo Point Cloud Dataset. The initial mesh is constructed using the Series of Local Triangulations (SOLT), followed by resampling based on the desired point-to-point distance criterion (Figure 8).

We demonstrate 50% and 75% reductions in point density while preserving the genus through reconstructed meshes. For refinement, Restricted Voronoi Diagram-based (RVD) re-meshing techniques [17] are suggested. The results (summarized in Table 2) show that the resampled point clouds maintain the genus and exhibit uniform density distribution due to constrained blue noise sampling.

### 3.3. Mechanical Geometries

**Figure 9:**
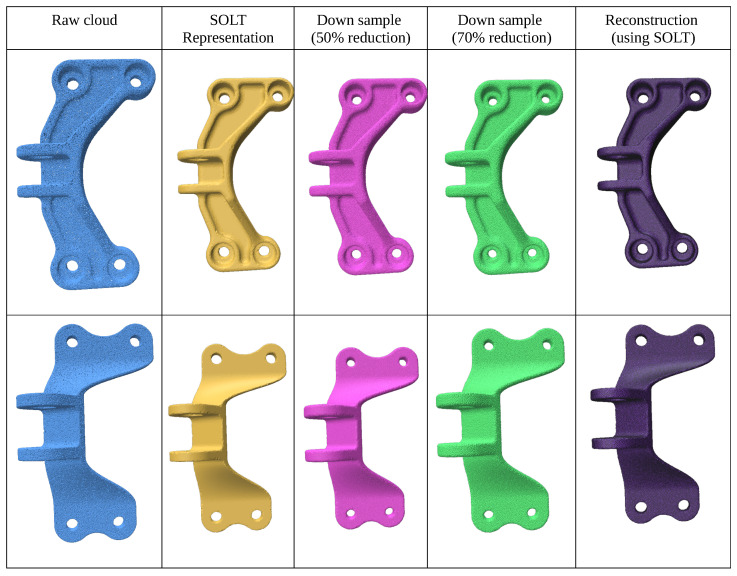
Point clouds synthesized from the SimJEB dataset. Point cloud (blue) meshed using SOLT (yellow), downsampled in two stages (pink and green), and reconstructed using the SOLT representation (purple).

This experiment evaluates geometries with intricate feature curves sampled from the SimJEB dataset [26]. Dense point clouds are generated using random point-cloud generation [27]. The results (summarized in Table 3) show effective feature preservation through explicit and implicit techniques (Figure 9).

### 3.4. Geometries with Intricate Features

To further evaluate the robustness of our algorithm, we tested intricate geometries sourced from the Thingi10k dataset [25]. These geometries include objects with sharp creases and mixed features, presenting significant challenges for resampling and reconstruction techniques. The geometries were first converted into point clouds from triangular meshes to serve as input for the resampling process.

The SOLT method was applied to generate an initial mesh representation of the point cloud, followed by blue noise sampling for resampling. Feature preservation was achieved using the feature distance field technique described in Section 2.5. This approach effectively retained sharp and smooth features, demonstrating the versatility and robustness of the algorithm.

**Figure 10:**
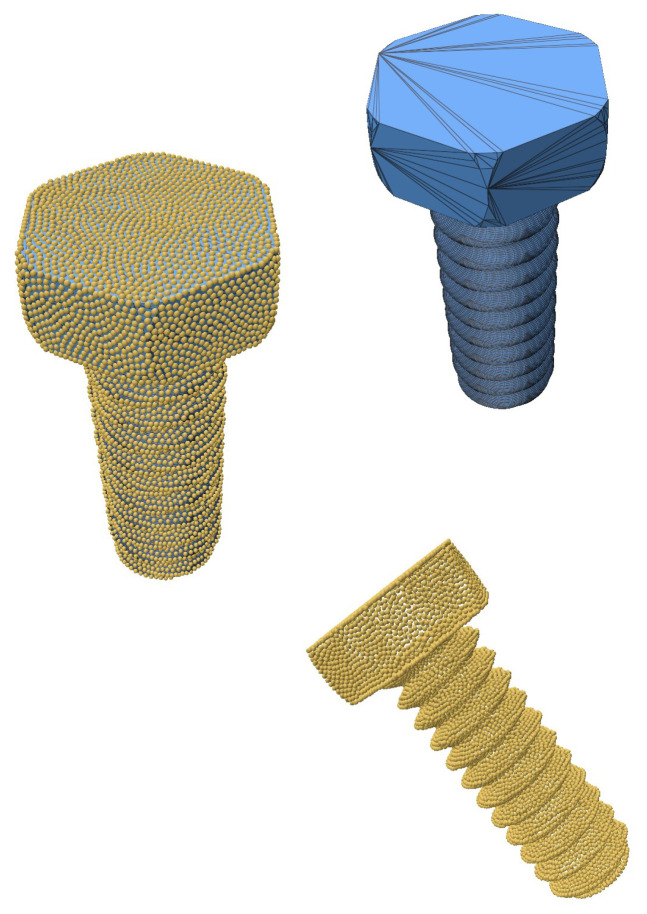
A screw geometry resampled using our algorithm (geometry from the Thingi10k dataset).

**Figure 11:**
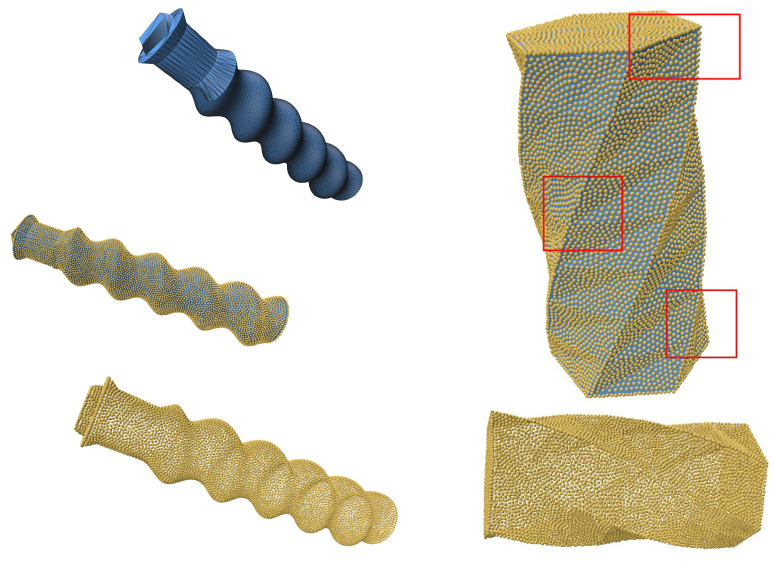
A mixture of smooth and sharp geometries with twist-like features (geometries from the Thingi10k dataset).

**Figure 12:**
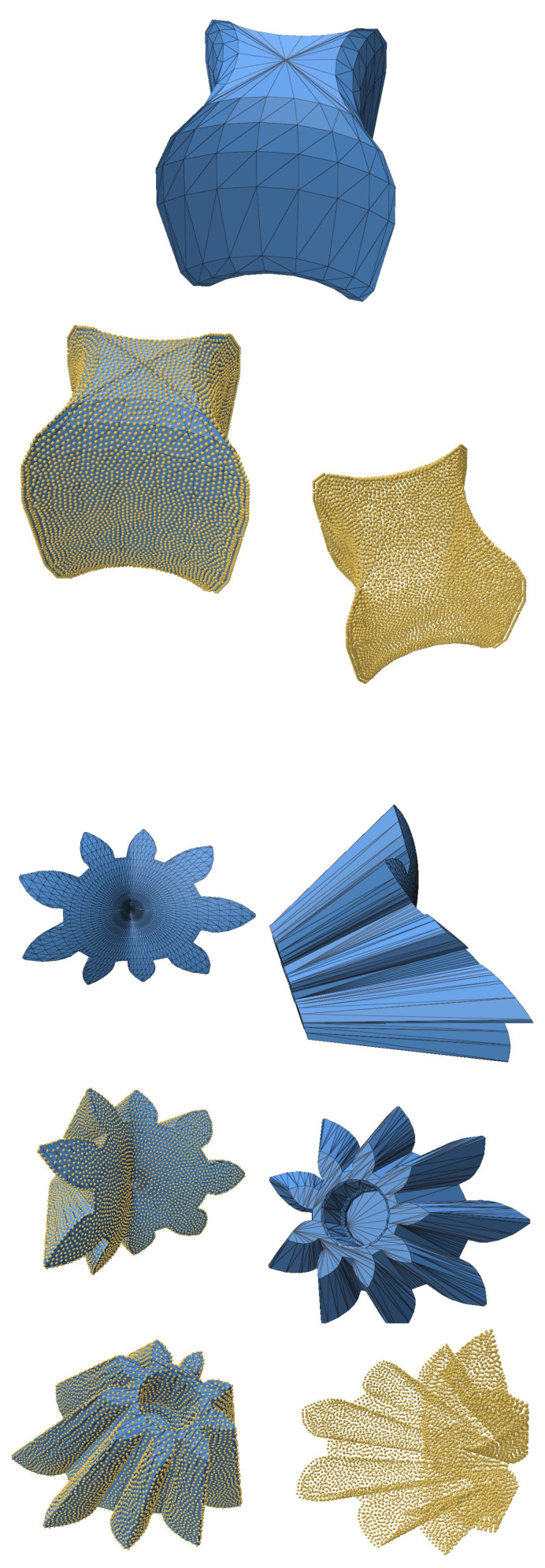
Mechanical components from the Thingi10k dataset. Sharp creases were recovered perfectly.

The results (summarized in Table 4), illustrated in Figure 10, Figure 11 and Figure 12, confirm that the algorithm accurately preserves intricate details such as sharp creases and twist-like features. These findings highlight the adaptability of the SOLT method to handle diverse geometric complexities.

#### Summary

The evaluation of intricate geometries highlights the effectiveness of the SOLT method in preserving fine details, such as sharp creases and mixed topological features. The algorithm demonstrates robustness across diverse scenarios, achieving Chamfer and Hausdorff distance losses within minimal ranges, while maintaining uniformity and volume preservation. The observed computational efficiency and compression ratios further validate the adaptability of this method to handle complex geometries.

### 3.5. Applications

The most straightforward application of our algorithm is surface reconstruction. For surfaces with defects, SOLT-based reconstruction followed by resampling and basic point-cloud meshing techniques can effectively repair problematic meshes.

Additionally, our method is a robust intermediate representation for preprocessing challenging geometries in intrinsic triangulations, ensuring better conditioning and improved mesh quality. With its flexibility and robustness, the algorithm can seamlessly integrate into existing workflows requiring mesh improvement, resampling, or defect handling.

**Figure 13:**
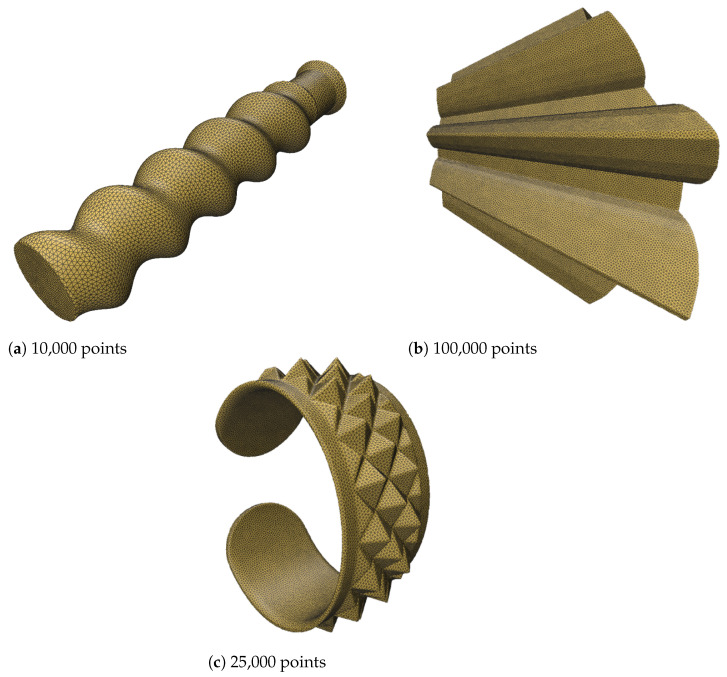
Selected geometries from the Thingi10k dataset, resampled using our algorithm and reconstructed using a simple Ball-Pivoting Algorithm (BPA) [28]. The results demonstrate the uniformity and quality of the reconstructed meshes.

**Figure 14:**
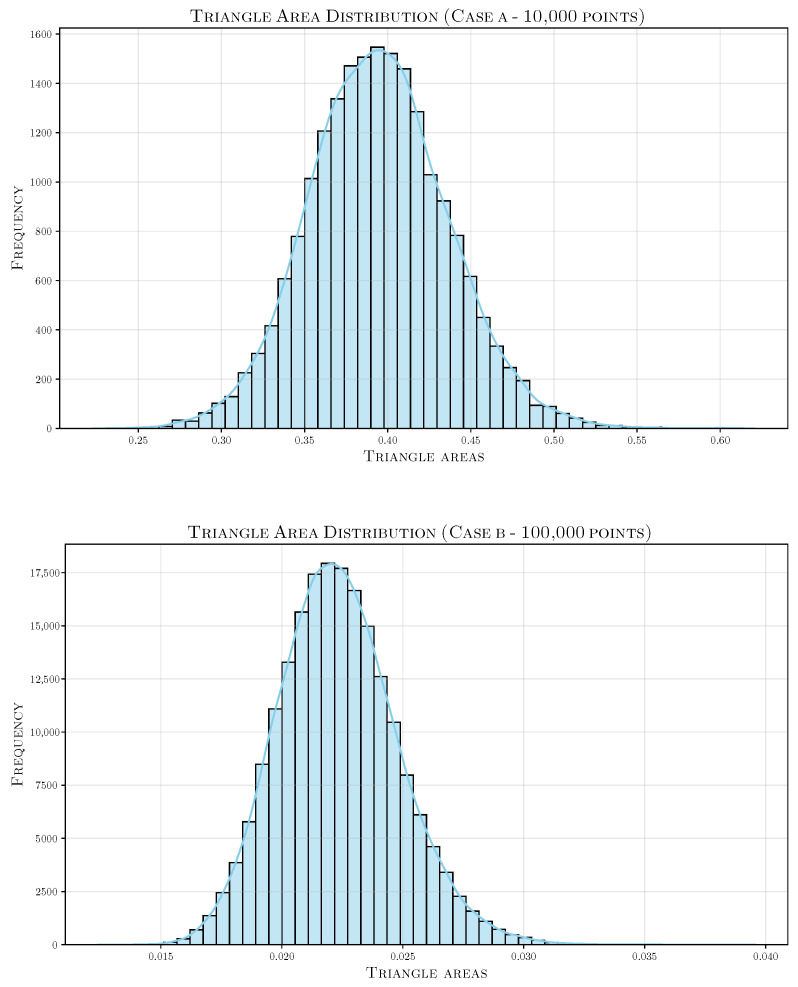

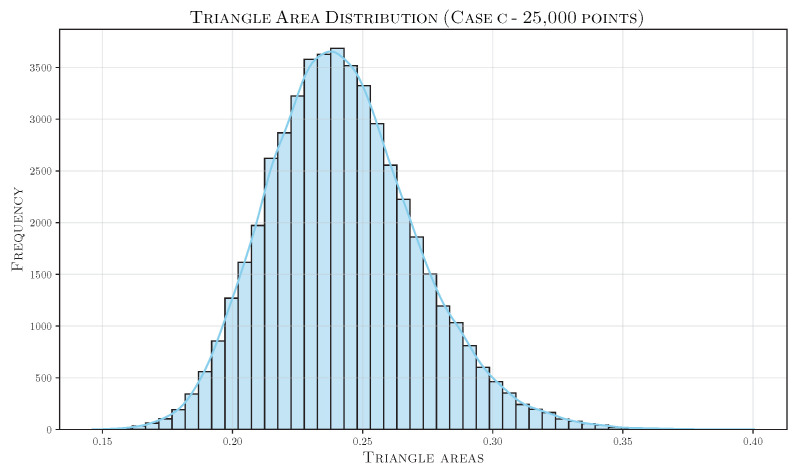
Histograms showing the triangle area distribution for the reconstructed geometries presented in Figure 13.

We showcase selected geometries from the Thingi10k dataset, resampled using our algorithm. These geometries were reconstructed using a simple Ball-Pivoting Algorithm (BPA) [28] (Figure 13). The triangle area histograms (Figure 14) for the reconstructed meshes highlight the uniformity achieved through our resampling approach, demonstrating the consistency of the method.

## 4. Comparison Against Existing Works

In this section, we compare the resampling capabilities of our algorithm against selected existing works from the literature. A small subset of examples is chosen for this comparison, focusing on those with readily available implementations and examples. Many existing works were excluded from this comparison as they either lack support for Linux or require specific hardware, such as high-end GPUs, which limits their accessibility.

### 4.1. Traditional Methods

The method proposed in [12] introduces a two-step framework for intrinsic and isotropic resampling. It combines efficient intrinsic control using geodesic measurements with geometrically optimized resampling to address challenges such as non-uniform point density and adjacency information in point clouds. This algorithm demonstrates strong performance in applications such as point-cloud simplification, mesh reconstruction, and shape registration, leveraging geometric updates for isotropic or adaptively isotropic resampling.

**Figure 15:**
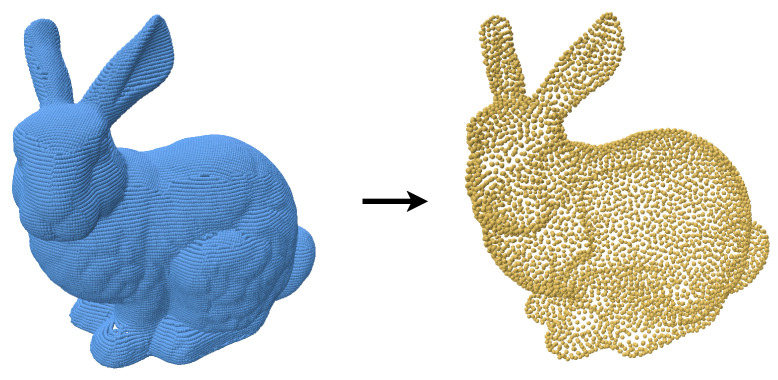
Input bunny point cloud along with a 5000-point resample produced by [12]. These results were provided by the authors.

**Figure 16:**
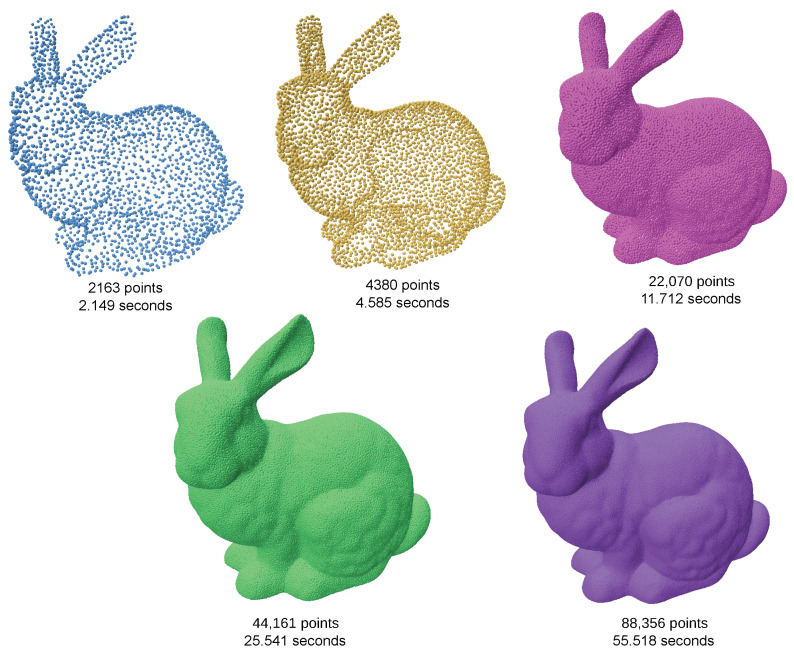
Bunny resampled at various sizes using SOLT, along with corresponding sampling times. The results demonstrate that SOLT maintains consistent efficiency and quality as sample size increases, comparable to the algorithms proposed in [12].

Despite its strengths, the efficiency of the algorithm in [12] significantly decreases when the target output exceeds 50,000 points (Figure 15). This is primarily due to the computational overhead of Delaunay triangulation and geodesic coordinate mapping, which impact its scalability for high-resolution point clouds. As a result, the algorithm is less practical for applications requiring large-scale resampling. Our method maintains its efficiency and quality irrespective of size of the target output (Figure 16).

### 4.2. Learning-Based Methods

The method proposed in [16], known as RepKPU, introduces a novel approach to point-cloud upsampling by leveraging kernel point representation and a Kernel-to-Displacement paradigm. This technique reformulates upsampling as the deformation of kernel points guided by learned geometric features, enabling density-sensitive and position-adaptive local geometry representations. RepKPU demonstrates superior performance across several benchmarks, including the PU-GAN and PU1K datasets, producing smoother and more uniform point clouds while maintaining computational efficiency.

**Figure 17:**
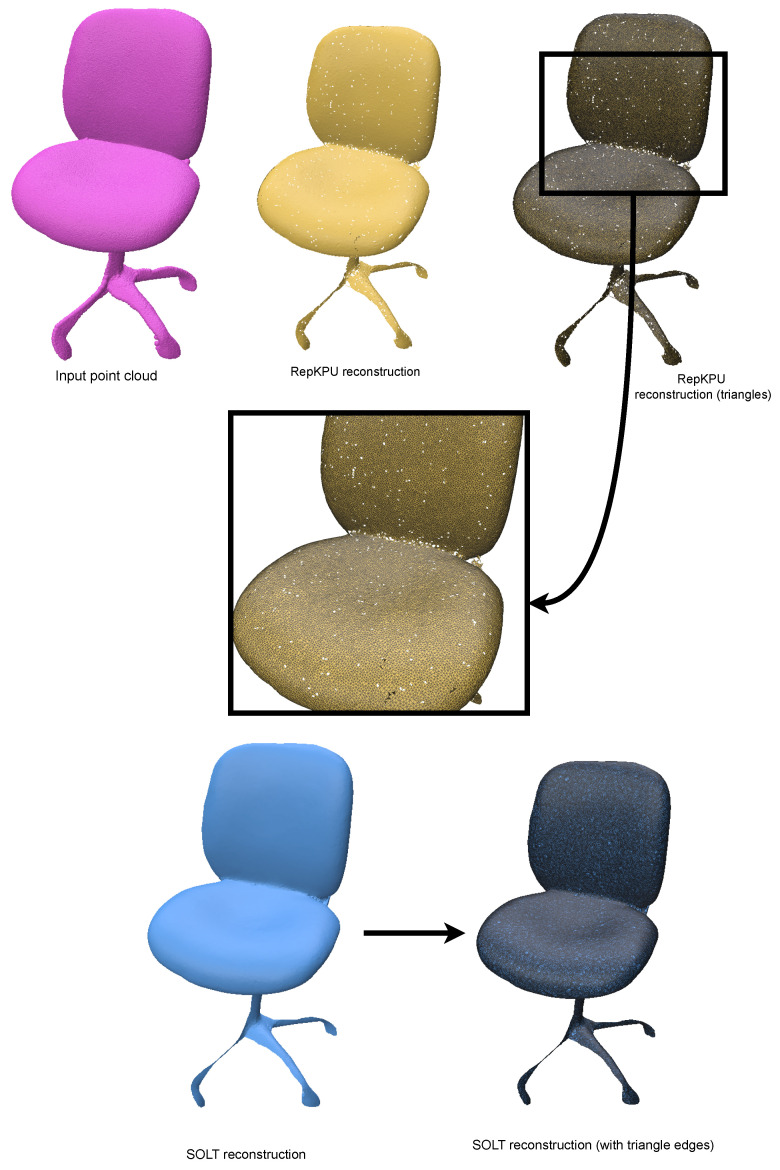
Chair reconstruction from input point cloud using the RepKPU workflow (results shared by the authors). The reconstruction contains multiple holes and is of poor quality. For comparison, the SOLT reconstruction of the same chair geometry is shown, demonstrating significantly higher quality and robustness.

**Figure 18:**
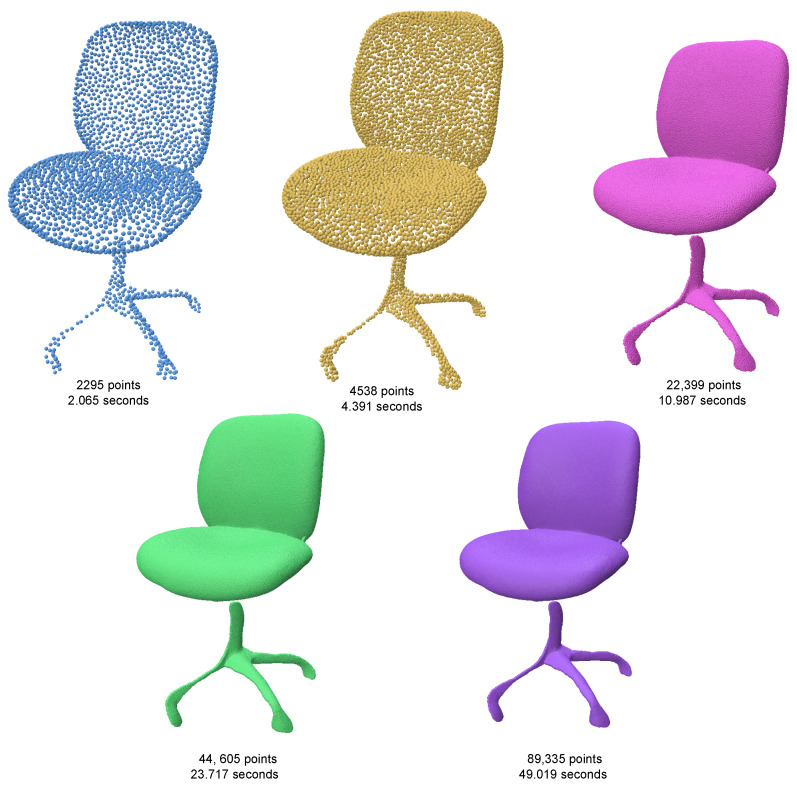
Chair resampled at various sizes using SOLT, along with corresponding sampling times. The results demonstrate that SOLT maintains consistent efficiency and high-quality output as the sample size increases.

For this study, we used the chair example provided by the authors of RepKPU (Figure 17). When tested on the same data, our method generated a hole-free reconstruction of significantly higher quality compared to RepKPU (Figure 18). This comparison underscores the robustness and effectiveness of our approach, particularly in scenarios demanding high-precision resampling.

## 5. Conclusions

We presented a novel approach for resampling point clouds based on the proposed intermediate representation, “Series of Local Triangulations" (SOLT). This method overcomes the limitations of voxelization-based techniques, offering a robust and flexible solution for both upsampling and downsampling.

Through extensive testing on point clouds derived from 3D scans and mechanical geometries, we demonstrated the effectiveness of our algorithm in preserving features, maintaining point-to-point distance criteria, and ensuring geometric fidelity. Additionally, we showcased the potential of our method as a surface reconstruction tool, particularly for repairing defective meshes and improving the quality of reconstructed surfaces.

This approach’s seamless integration into existing CAE workflows makes it a valuable tool for practitioners in academic and industrial settings. Our method advances state-of-the-art techniques in point-cloud processing and related applications by addressing challenges in feature preservation, surface reconstruction, and geometric fidelity.

## 6. Limitations

The algorithms proposed in this paper are specifically designed and extensively tested for CAE workflows, with examples tailored to this domain. Consequently, their robustness and performance in other fields may not align with the levels demonstrated in our experiments. Adapting and enhancing these algorithms for broader applications remains an open challenge for future research.

Additionally, the current implementation does not incorporate parallelization in any part of the algorithmic workflow. Introducing parallel processing and further code optimizations could significantly improve both the speed and robustness of the existing algorithms, making them more suitable for large-scale and time-sensitive applications.

## Figures and Tables

**Table 1:** Comparison of SOLT, BPA, and Poisson reconstruction.

Criterion	SOLT	BPA	Poisson
Reconstruction Time (Eagle Example)	Fast (35.8 s)	Very Slow (26.91 min)	Moderate (87.9 s)
Accuracy	High	High	High
Suitability for Resampling	Excellent	Moderate	Poor
Requires Surface Normals	No	Yes	Yes
Suitability for PDE Solvers	Good	Moderate	Excellent
Computational Efficiency	Very High	Low	Moderate
Topology Preservation	Excellent	Good	May alter topology
Feature Sensitivity	Preserves fine details	Sensitive to noisy data	Over-smoothing of features is common
Scalability for Large Datasets	Highly Scalable	Poor	Moderate—Some optimized variants exist

**Table 2:** Quantitative metrics for smooth geometries.

Metric	Range (% or Value)
Chamfer Distance Loss (%)	0.1–0.3
Hausdorff Distance Loss (%)	0.22–1.25
Uniformity Index (%)	96–99
Volume Preservation Error (%)	1.5
Computational Time (s)	5.0–30.0
Compression Ratio	2:1–3.33:1

**Table 3:** Quantitative metrics for mechanical geometries.

Metric	Range (% or Value)
Chamfer Distance Loss (%)	0.3–0.6
Hausdorff Distance Loss (%)	0.8–1.3
Uniformity Index (%)	97–99
Volume Preservation Error (%)	0.5–1.0
Computational Time (s)	7.5–47.9
Compression Ratio	2:1–3.33:1

**Table 4:** Quantitative metrics for intricate feature geometries.

Metric	Observed Range (% or Value)
Chamfer Distance Loss (%)	0.45–0.78
Hausdorff Distance Loss (%)	0.92–1.6
Uniformity Index (%)	96–97.5
Volume Preservation Error (%)	1.2–1.8
Computational Time (s)	12.0–46.2
Compression Ratio	2.5:1–3.33:1

## Data Availability

We used the Waterloo point cloud database, SimJEB dataset, Thingi10k dataset and Eagle geometry from the Open3D dataset.

## References

[1] Rios T., Wollstadt P., Stein B.V., Back T., Xu Z., Sendhoff B., Menzel S. Scalability of Learning Tasks on 3D CAE Models Using Point Cloud Autoencoders. Proceedings of the 2019 IEEE Symposium Series on Computational Intelligence (SSCI).

[2] Fornberg B., Flyer N. (2015). A Primer on Radial Basis Functions with Applications to the Geosciences.

[3] Suchde P., Jacquemin T., Davydov O. (2023). Point Cloud Generation for Meshfree Methods: An Overview. Arch. Comput. Methods Eng..

[4] Aliev K.A., Sevastopolsky A., Kolos M., Ulyanov D., Lempitsky V. (2020). Neural Point-Based Graphics. arXiv.

[5] Metzer G., Hanocka R., Zorin D., Giryes R., Panozzo D., Cohen-Or D. (2021). Orienting Point Clouds with Dipole Propagation. ACM Trans. Graph..

[6] Deng Q., Zhang S., Ding Z. (2022). An Efficient Hypergraph Approach to Robust Point Cloud Resampling. IEEE Trans. Image Process..

[7] Chen S., Tian D., Feng C., Vetro A., Kovačević J. (2018). Fast Resampling of Three-Dimensional Point Clouds via Graphs. IEEE Trans. Signal Process..

[8] Lv C., Lin W., Zhao B. (2023). Intrinsic and Isotropic Resampling for 3D Point Clouds. IEEE Trans. Pattern Anal. Mach. Intell..

[9] Xiao Y., Zhang T., Cao J., Chen Z. (2024). Accelerated Lloyd’s Method for Resampling 3D Point Clouds. IEEE Trans. Multimed..

[10] Jiao X., Lv C., Zhao J., Yi R., Wen Y.H., Pan Z., Wu Z., Liu Y.J. (2025). Weighted Poisson-disk Resampling on Large-Scale Point Clouds. arXiv.

[11] Fei B., Yang W., Chen W.M., Li Z., Li Y., Ma T., Hu X., Ma L. (2022). Comprehensive Review of Deep Learning-Based 3D Point Cloud Completion Processing and Analysis. IEEE Trans. Intell. Transp. Syst..

[12] Zhao L., Hu Y., Yang X., Dou Z., Kang L. (2024). Robust multi-task learning network for complex LiDAR point cloud data preprocessing. Expert Syst. Appl..

[13] Zhao L., Hu Y., Yang X., Dou Z., Wu Q. (2025). ICDDPM: Image-conditioned denoising diffusion probabilistic model for real-world complex point cloud single view reconstruction. Expert Syst. Appl..

[14] Wu C., Zheng J., Pfrommer J., Beyerer J. Attention-Based Point Cloud Edge Sampling. Proceedings of the IEEE/CVF Conference on Computer Vision and Pattern Recognition (CVPR).

[15] Chen H., Du B., Luo S., Hu W. (2022). Deep Point Set Resampling via Gradient Fields. IEEE Trans. Pattern Anal. Mach. Intell..

[16] Rong Y., Zhou H., Xia K., Mei C., Wang J., Lu T. RepKPU: Point Cloud Upsampling with Kernel Point Representation and Deformation. Proceedings of the IEEE/CVF Conference on Computer Vision and Pattern Recognition.

[17] Suriyababu V.K., Vuik C., Möller M. (2023). Towards a High Quality Shrink Wrap Mesh Generation Algorithm Using Mathematical Morphology. Comput.-Aided Des..

[18] Zhou Q.Y., Park J., Koltun V. (2018). Open3D: A Modern Library for 3D Data Processing. arXiv.

[19] Sharp N., Crane K. (2020). A Laplacian for Nonmanifold Triangle Meshes. Comput. Graph. Forum (SGP).

[20] Xu R., Dou Z., Wang N., Xin S., Chen S., Jiang M., Guo X., Wang W., Tu C. (2023). Globally Consistent Normal Orientation for Point Clouds by Regularizing the Winding-Number Field. ACM Trans. Graph. (TOG).

[21] Bridson R. (2007). Fast Poisson Disk Sampling in Arbitrary Dimensions. Proceedings of the ACM SIGGRAPH 2007 Sketches.

[22] Feng N., Crane K. (2024). A Heat Method for Generalized Signed Distance. ACM Trans. Graph..

[23] Su H., Duanmu Z., Liu W., Liu Q., Wang Z. Perceptual quality assessment of 3D point clouds. Proceedings of the 2019 IEEE International Conference on Image Processing (ICIP).

[24] Liu Q., Su H., Duanmu Z., Liu W., Wang Z. (2022). Perceptual Quality Assessment of Colored 3D Point Clouds. IEEE Trans. Vis. Comput. Graph..

[25] Zhou Q., Jacobson A. (2016). Thingi10K: A Dataset of 10,000 3D-Printing Models. arXiv.

[26] Whalen E., Beyene A., Mueller C. (2021). SimJEB: Simulated Jet Engine Bracket Dataset. Comput. Graph. Forum.

[27] Turk G. (1990). Generating Random Points in Triangles. Graphics Gems.

[28] Bernardini F., Mittleman J., Rushmeier H., Silva C., Taubin G. (1999). The ball-pivoting algorithm for surface reconstruction. IEEE Trans. Vis. Comput. Graph..

